# A pseudokinase couples signaling pathways to enable asymmetric cell division in a bacterium

**DOI:** 10.15698/mic2015.01.184

**Published:** 2014-12-30

**Authors:** W. S. Childers, Lucy Shapiro

**Affiliations:** 1 Department of Developmental Biology, Stanford University School of Medicine, Stanford, California, 94305.

**Keywords:** Caulobacter crescentus, histidine kinase, two-component systems (TCS), response regulator, pseudokinase, asymmetric cell division, cell-cycle

## Abstract

Bacteria face complex decisions when initiating developmental events such as sporulation, nodulation, virulence, and asymmetric cell division. These developmental decisions require global changes in genomic readout, and bacteria typically employ intricate (yet poorly understood) signaling networks that enable changes in cell function. The bacterium *Caulobacter crescentus* divides asymmetrically to yield two functionally distinct cells: a motile, chemotactic swarmer cell, and a sessile stalked cell with replication and division capabilities. Work from several *Caulobacter* labs has revealed that differentiation requires concerted regulation by several two-component system (TCS) signaling pathways that are differentially positioned at the poles of the predivisional cell (Figure 1). The strict unidirectional flow from histidine kinase (HK) to the response regulator (RR), observed in most studied TCS, is difficult to reconcile with the notion that information can be transmitted between two or more TCS signaling pathways. In this study, we uncovered a mechanism by which daughter cell fate, which is specified by the DivJ-DivK-PleC system and effectively encoded in the phosphorylation state of the single-domain RR DivK, is communicated to the CckA-ChpT-CtrA signaling pathway that regulates more than 100 genes for polar differentiation, replication initiation and cell division. Using structural biology and biochemical findings we proposed a mechanistic basis for TCS pathway coupling in which the DivL pseudokinase is repurposed as a sensor rather than participant in phosphotransduction.

## Going beyond two-components to regulate bacterial cell-fate

**Figure 1:**
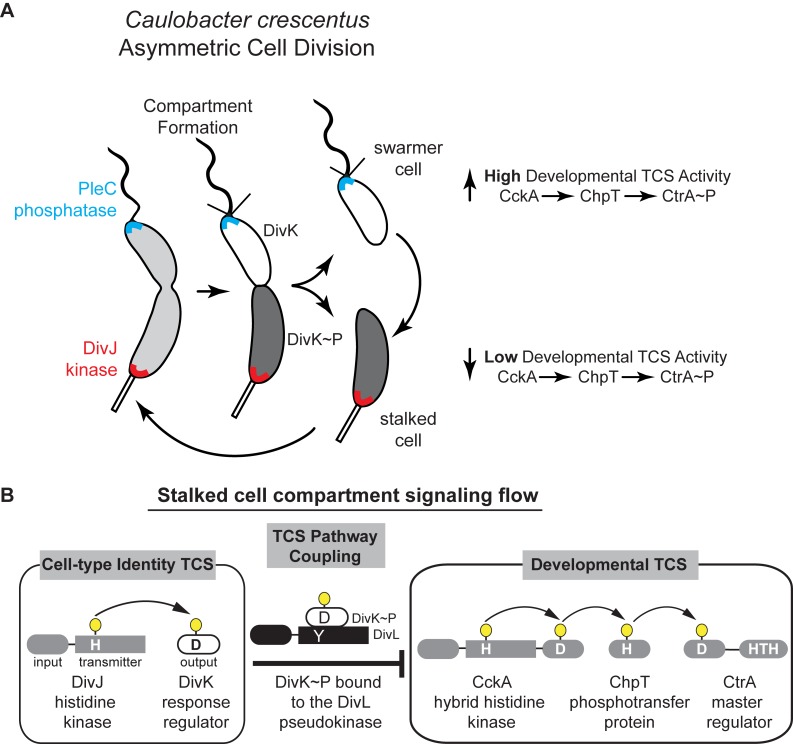
FIGURE 1: **(A)** Asymmetric cell division in *Caulobacter crescentus* is driven by a single-domain response regulator (DivK~P, shaded gray within cell cartoons) that has been inversely correlated with the activity of a cell-cycle kinase (CckA) that in turn activates the CtrA transcription factor regulating over 100 developmental genes. **(B)** The kinase DivJ phosphorylates DivK in the stalked compartment, while PleC dephosphorylates DivK in the swarmer compartment. The pseudokinase DivL forms a complex with DivK~P and couples these two signaling pathways.

More than two decades of research from the labs of Christine Jacobs-Wagner, Michael Laub, Kathleen Ryan, Yves Brun, Patrick Viollier, Sean Crosson, Urs Jenal, Emanuele Biondi, Austin Newton, and our own have revealed critical roles for TCSs in the regulation of *Caulobacter's* asymmetric cell-division. Among them are the DivJ-DivK-PleC pathway, which establishes cell identity through the unequal partitioning of a kinase (DivJ) at one cell pole and a phosphatase (PleC) at the other cell pole (Figure 1). Differential positioning of these signal transduction proteins upon compartmentalization prior to the completion of cell division enables cell-type specific phosphorylation of the DivK response regulator (RR) (Figure 1). A second critical TCS system, CckA-ChpT-CtrA pathway, is a phosphorelay system that controls activation of the CtrA master transcription regulator CtrA that regulates polar differentiation and cell-cycle progression. An unusual histidine kinase, DivL, was shown by the Laub and Ryan labs to bind DivK~P specifically and inhibit the CckA pathway *in vivo *by a poorly defined mechanism (Figure 1).

## Loss of HK catalytic functions results in response regulator sensor

Structural analysis in collaboration with Ashley Deacon and Qingping Xu revealed that DivL adopts a HK structural fold, which normally functions by regulating the phosphorylation state of a response regulator. In contrast, we found that DivL does not covalently modify the phosphorylation state of DivK. Instead, we observed that DivL preferentially binds phosphorylated DivK (DivK~P) over the unphosphorylated form. Additionally, the phosphorylation state of DivK~P is significantly stabilized in the presence of DivL. These observations suggest that the non-catalytic kinase output domain of DivL has been uniquely converted into aninput sensory domain that specifically detects the phosphorylated form of the RR DivK.

**Figure 2:**
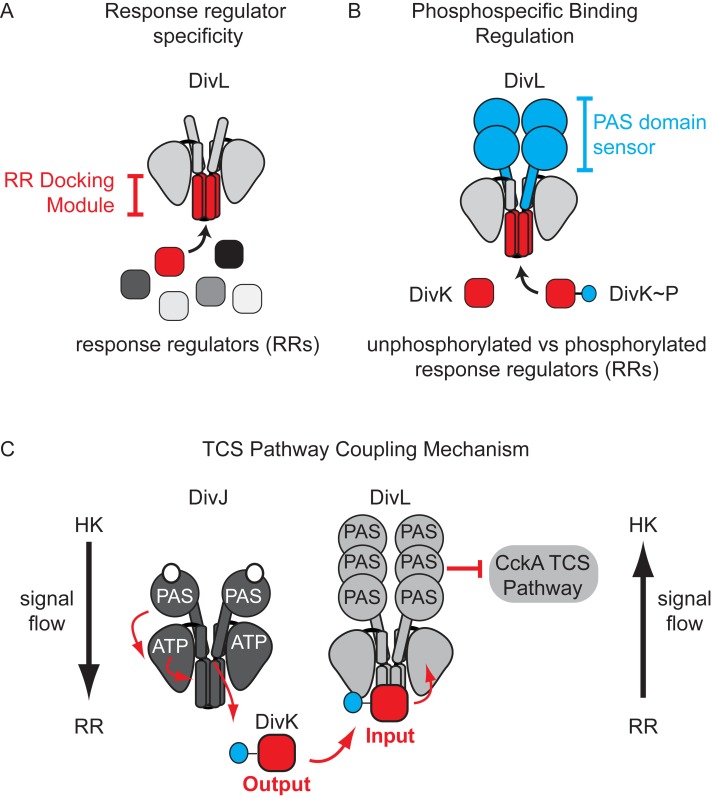
FIGURE 2: Factors that program complex signal wiring of two-component systems. **(A)** Previous work has shown that the protein-protein interaction surface within the RR docking module selects the response regulator phosphotransfer partner. **(B)** We observed that the PAS sensory domain plays a role in phosphospecific recognition of RR~P over unphosphorylated RR. **(C)** Signaling flow from the response regulator DivK~P to the CckA TCS signaling pathway occurs via the pseudo-histidine kinase DivL. Unlike typical histidine kinases, in which signal flows from an HK to an RR, the pseudokinase DivL receives signaling information by binding to its cognate RR (DivK~P), which is then transmitted to the kinase CckA. This reversal of the typical TCS signaling flow enables coupling of two TCS systems that promote asymmetric cell division.

A key question we addressed in this study was how DivL specifically binds DivK~P over DivK. Many laboratories have convincingly demonstrated that surface-exposed residues within the “RR docking module” of a TCS HK specify the binding between a given HK and its cognate RR (Figure 2A), and we indeed found that a set of RR docking module point mutations in DivL disrupt DivK binding and, consequently, result in a loss of cell viability. However, the contribution of PAS family sensor domains to the HK-RR interaction is less well understood. We critically observed that DivL variants containing *only* the kinase region bound similarly to both the unphosphorylated and phosphorylated forms of DivK. Only when the sensory PAS domain was added back to this "kinase-only" DivL mutant did we observe highly specific recognition of DivK~P over unphosphorylated DivK (Figure 2B). Taken together, our findings suggest that the RR docking region and the attached sensory domain cooperate to not only specify the RR binding partner but also to selectively detect covalent modifications made to the cognate RR (Figure 2C). We propose that these observations could have broad implications for how TCS kinases can influence whether a protein containing a HK-fold functions as a kinase by promoting unphosphorylated RR binding or as a phosphatase by promoting RR~P binding. Future structural characterization will dissect whether the PAS sensory domain influence on the HK-RR interaction occurs by reconfiguring RR docking region or by directly making contacts with the RR.

## A reversal of signaling flow enables TCS pathway coupling

Work from the Laub and Ryan labs revealed that *in vivo* DivL functions as a cell-fate switch by *inhibiting* CckA signaling activity when bound to DivK~P. To understand how DivK~P binding imparts a switch-like function to DivL (Figure 1) we examined a DivL structural region that is broadly important for allosteric kinase activation. Despite its physical distance from the RR docking module, we found that a point mutation within this DivL allosteric regulatory region eliminated binding of DivK~P to DivL in a PAS domain dependent manner. From these experiments, we propose that DivK~P binding to DivL drives a conformational change within the DivL PAS sensory domain to allow a DivL-CckA interaction that inhibits the CckA signaling pathway (Figure 2C). Therefore, unlike conventional HK-RR signaling pathways, the signaling information in this case flows in reverse direction - from the RR DivK to the pseudokinase DivL (Figure 2C). Critically, this signal flow reversal connects two TCS pathways that culminate in the regulation of differential gene expression in daughter cells that result from asymmetric cell division. This finding raises another important question: do other bacterial TCS signaling systems take advantage of reversals in signal flow?

## Pseudokinases in Bacteria

While eukaryotic pseudokinases have been demonstrated to play crucial roles in signaling, few studies of bacterial TCS pseudokinases have been reported. Loss of DivL kinase catalytic functions allows other functional capabilities within the HK-fold to emerge. We have shown that the pseudokinase DivL, with a tyrosine replacing the histidine, retains one HK-fold function in that it binds a cognate RR; our results also identified a second conserved HK function, namely the ability to undergo switch-like conformational change upon binding to a phosphorylated RR. Conservation of these two functions uniquely re-wires DivL to connect two TCS signaling pathways. More generally, the pseudokinase DivL shares broad characteristics with eukaryotic pseudokinases: (1) DivL modulates the signaling activity of another functional kinase (CckA), and (2) other work led by Antonio Iniesta has shown that DivL is necessary to recruit CckA to the signaling hub at the new cell pole. Approximately 1% of sequenced TCS HKs contain an amino acid substitution at the phosphorylatable histidine site (e.g. Tyr, Gln, Asn, Arg, Asp), raising the possibility that bacteria utilize pseudokinases where simple TCS pathways offer inadequate processing capabilities.

A second interesting trend in bacterial developmental TCS signaling pathways is the prevalence of histidine or pseudohistidine kinases with multi-PAS domain sensors. For example, the *Caulobacter *genome alone encodes more than a dozen multi-PAS-domain sensor kinases. Multiple PAS sensory domains offer the potential to integrate multiple signals, although very little is known about how multi-domain sensors integrate information. Are these sensory domains independent of one another, functioning as OR logic gates, or do they work cooperatively as AND gates? Given that PAS sensory domains are commonly involved in protein-protein interactions, it will be interesting to determine whether signaling cross-talk occurs between other HK sensory domains. Future discovery of bacterial pseudokinases and dissections of HK sensory domain mechanisms will help illuminate how bacteria have modified and adapted the canonical HK-fold to generate complex signaling circuits that underlie critical developmental decisions.

